# Validating subcellular localization prediction tools with mycobacterial proteins

**DOI:** 10.1186/1471-2105-10-134

**Published:** 2009-05-07

**Authors:** Daniel Restrepo-Montoya, Carolina Vizcaíno, Luis F Niño, Marisol Ocampo, Manuel E Patarroyo, Manuel A Patarroyo

**Affiliations:** 1 Fundación Instituto de Inmunología de Colombia (FIDIC), Carrera 50 No, 26-20 Bogotá DC, Colombia; 2 Intelligent Systems Research Laboratory (LISI), Universidad Nacional de Colombia, Carrera 45 No, 26-85, Bogotá DC, Colombia; 3 Research Group on Combinatorial Algorithms (ALGOS-UN), Universidad Nacional de Colombia, Carrera 45 No, 26-85, Bogotá DC, Colombia; 4 School of Medicine, Universidad del Rosario, Carrera 24 No, 63C-69, Bogotá DC, Colombia; 5 School of Medicine, Universidad Nacional de Colombia, Carrera 45 No 26-85, Bogotá DC, Colombia

## Abstract

**Background:**

The computational prediction of mycobacterial proteins' subcellular localization is of key importance for proteome annotation and for the identification of new drug targets and vaccine candidates. Several subcellular localization classifiers have been developed over the past few years, which have comprised both general localization and feature-based classifiers. Here, we have validated the ability of different bioinformatics approaches, through the use of SignalP 2.0, TatP 1.0, LipoP 1.0, Phobius, PA-SUB 2.5, PSORTb v.2.0.4 and Gpos-PLoc, to predict secreted bacterial proteins. These computational tools were compared in terms of sensitivity, specificity and Matthew's correlation coefficient (MCC) using a set of mycobacterial proteins having less than 40% identity, none of which are included in the training data sets of the validated tools and whose subcellular localization have been experimentally confirmed. These proteins belong to the TBpred training data set, a computational tool specifically designed to predict mycobacterial proteins.

**Results:**

A final validation set of 272 mycobacterial proteins was obtained from the initial set of 852 mycobacterial proteins. According to the results of the validation metrics, all tools presented specificity above 0.90, while dispersion sensitivity and MCC values were above 0.22. PA-SUB 2.5 presented the highest values; however, these results might be biased due to the methodology used by this tool. PSORTb v.2.0.4 left 56 proteins out of the classification, while Gpos-PLoc left just one protein out.

**Conclusion:**

Both subcellular localization approaches had high predictive specificity and high recognition of true negatives for the tested data set. Among those tools whose predictions are not based on homology searches against SWISS-PROT, Gpos-PLoc was the general localization tool with the best predictive performance, while SignalP 2.0 was the best tool among the ones using a feature-based approach. Even though PA-SUB 2.5 presented the highest metrics, it should be taken into account that this tool was trained using all proteins reported in SWISS-PROT, which includes the protein set tested in this study, either as a BLAST search or as a training model.

## Background

The computational prediction of protein subcellular localization has been an important task accomplished by bioinformatics and many computational tools have been developed over the last two decades for this purpose [1-3]. Bioinformatics tools have largely been based on machine-learning methods such as artificial neural networks (ANNs), hidden Markov models (HMMs) and support vector machines (SVMs) [3]; all of which share the common feature of being data driven, ie, they can be trained based on examples and further optimized [2,4].

Protein trafficking and localization to the cell membrane in prokaryotic cells is mainly mediated by a translocation machinery that specifically recognizes a signal peptide at the protein's N-terminus [5,6], which is commonly referred to as the classical secretory pathway or the sec-dependent pathway [2,7]. However, a large number of proteins that are expressed on the cell surface or are secreted to the cell milieu do not have an intrinsic signal peptide, and hence are grouped as proteins transported via non-classical secretory pathways [8]. There are also other mechanisms alternative to the classical secretory pathway by which proteins having consensus motifs within their signal peptides are secreted [4]; such mechanisms include twin arginine translocation (Tat) and lipoprotein transport pathways.

Several studies have been carried out with the common goal of comparing the general predictive values of different computational tools, in terms of specificity and sensitivity percentages. In this work, we have validated the ability of two types of machine-learning tools to predict bacterial secreted proteins: a feature-based approach for which we used SignalP 2.0 [9], TatP 1.0 [10], LipoP 1.0 [11] and Phobius [12], and a general localization approach for which we used PA-SUB 2.5 included in Proteome Analyst 3.0 [1], Gpos-PLoc [13] and PSORTb v.2.0.4 [14]. Such tools are well known for their high performance in predicting signal peptide, protein subcellular localization and characteristic motifs displayed by transmembrane proteins.

Given the need for reliable computational tools suitable to predict Gram-positive secreted proteins and the inherent difficulty in isolating mycobacterial surface proteins *in vitro *due to the envelope's intrinsic complexity [15], we have validated the above mentioned tools based on a set of 272 mycobacterial proteins having less than 40% identity, as assessed in this study by comparing dipeptides with the Cd-hit algorithm [16,17]. Such protein set comprises the data set of TBpred, a computational tool specifically designed to predict subcellular localization of mycobacterial proteins.

Our goal was to establish which tools predicted protein subcellular localization with higher accuracy and therefore which ones could be used to specifically identify mycobacterial secretory proteins, considering the high relevance of such kind of proteins in host-cell recognition and pathogenesis. For example, the use of bioinformatics tools can greatly improve the identification and characterization of possible candidates to be included in the design of a minimal-subunit based vaccine against tuberculosis [18], a disease that annually causes 9.2 million new cases and the death of approximately 1.7 million people worldwide (including HIV-infected people) [19]. Furthermore, knowledge regarding protein subcellular localization and the mechanisms mediating protein trafficking to cell membrane is of key relevance since it can further help improve such identification process.

## Results

### Data set

The Cd-hit algorithm *n *= 2 (where *n *denotes word length) was used to calculate protein identity in 852 proteins comprising the entire TBpred training data set [17], setting the identity threshold at 0.4 [16,20]. This analysis yielded a final validation set of 272 proteins consisting of: 26 proteins attached to membrane by lipid anchor (amla), 68 cytoplasmatic (cyto), 174 integral membrane proteins (imp) and 4 secreted proteins (sec).

### Evaluation metrics

There are at least four different ways to calculate protein identity [20], of which the mechanism used in this work was the one based on the length of the shortest sequence. This method clusters proteins into subsets sharing a similarity threshold set by the user, known as the sequence identity, and gives a representative sequence for each cluster.

According to the results obtained after determining the identity of the 852 sequences comprising the TBpred protein training set, only 340 non-redundant proteins were identified within the data set by the Cd-hit algorithm. However, when these proteins were compared against each tool's data sets, 68 proteins were present in both data sets and thus were removed from the test set to make sure that the validation set was completely independent. As a result, the final test set contained a total of 272 proteins. In particular, Mamoon *et al*. [17] obtained 343 proteins by using the same algorithm, however it should be noted that the algorithm's results might vary depending on the identity threshold chosen by the user.

The results obtained for the feature-based tools (SignalP 2.0, TatP 1.0 and LipoP 1.0) were considered as a single group in the analysis, same as reported by Gardy *et al*. [1]. This approach excluded proteins secreted via non-classical pathways from the group, since the validation aimed at comparing only proteins containing signal peptides or displaying characteristic secretion motifs. Even though LipoP 1.0 predicts type I and II Signal Peptidase (SPI and SPII) recognition sequences, Transmembrane Domains (TM) (indicative of secretion) or Cytoplasmatic Proteins (Cyt), only those proteins predicted as being cleaved by a Signal Peptidase II (SPII) were included in the present analysis, given that Juncker *et al*. argue that this tool was designed exclusively for identifying such type of proteins [11].

Once the final validation data set consisting of 204 secreted and 68 non-secreted mycobacterial proteins had been defined, we calculated the number of true positives (TPs), true negatives (TNs), false positives (FPs) and false negatives (FNs) obtained by each tool and used these values to construct a confusion matrix for each tool (see additional file 1) in order to determine which tools yielded higher predictive precision. The number of proteins identified within each parameter is shown in Table 1.

**Table 1:** Comparison between feature-based and general localization tools according to the validation metrics.

	**Feature-based tools**			**General localization tools**		
**Parameters**	**SignalP 2.0, TatP 1.0, LipoP 1.0**	**SignalP 2.0**	**Phobius**	**PA-SUB 2.5**	**Gpos-PLoc**	**PSORTb v.2.0.4**
**TPs**	91	87	55	204	147	145
**TNs**	65	65	64	68	53	56
**FPs**	3	3	4	0	14	1.0
**FNs**	113	117	149	0	57	14
**Specificity**	0.97	0.97	0.93	1.0	0.91	0.99
**Sensitivity**	0.45	0.43	0.27	1.0	0.72	0.91
**MCC**	0.37	0.35	0.22	1.0	0.45	0.84

	**Summatory**					

**Secreted (TPs+FNs)**	204	204	204	204	204	159
**Non secreted (TNs+FPs)**	68	68	68	68	67	57

**TPs**, True Positives. **TNs**, True Negatives. **FPs**, False Positives. **FNs**, False Negatives. **Secreted proteins (n = 204), non-secreted proteins (n = 68), N = 272**

The results show that from the group of feature-based tools, the combination between SignalP 2.0, TatP 1.0 and LipoP 1.0 recognized the largest number of TPs and FNs (being the latter value even higher). PA-SUB 2.5 was the general localization tool that reported the highest number of TPs, which varied between 55 and 204, being 204 the maximum possible value (total number of secreted proteins). On the other hand, the number of TNs varied less than the aforementioned parameter (range 53–68, maximum possible value = 68), being again PA-SUB 2.5 the tool recognizing the entire protein input set. The number of FPs also displayed a moderate variation (range 0–14, maximum possible = 68), while the number of FNs ranged between 0–149 (maximum possible value = 204), being the second parameter the one varying the most among all prediction tools.

### Specificity, Sensitivity and Matthews' Coefficient Correlation

According to the specificity results (where a 95% precision indicates that 5 out of every 100 proteins are FPs), in general, all tools presented high specificity, being PA-SUB 2.5, v.2.0.4, SignalP 2.0 and the combination between SignalP 2.0, TatP 1.0 and LipoP 1.0, the ones presenting specificity values closer to one.

Sensitivity, understood as the method's capacity to identify TPs (where a 95% sensitivity indicates that 5 out of every 100 proteins are FNs), displayed an interesting behavior. Only two tools had sensitivity values above 90% (PA-SUB 2.5 and PSORTb v.2.0.4).

MCC was used as a performance predictive metric as it estimates the TP rate of each tool by simultaneously incorporating precision and sensitivity. As shown in Table 1, MCC values for PA-SUB 2.5 and PSORTb v.2.0.4 varied around 90%, which indicates that these two algorithms have high performance to predict mycobacterial protein subcellular localization. Similarly, SignalP 2.0 was the most sensitive and precise feature-based tool when analyzed independently, as it yielded a higher MCC value than the one obtained by SignalP 2.0 in combination with TatP 1.0 and LipoP 1.0.

### Particularities in the predictions

Interestingly, TatP 1.0 identified 9 proteins having the Tat characteristic feature, four of which were predicted only by this tool, while the remaining ones were also identified by SignalP 2.0. On the contrary, LipoP 1.0 did not recognize any of the proteins in the input set as being SPII, which diminished this tool's TP value to zero.

With regard to general localization tools, some proteins were reported as "unknown", specifically Gpos-PLoc reported a protein within the non-secreted protein set, while PSORTb v.2.0.4 labeled 44 secreted and 11 non-secreted proteins as "unknown" (see additional file 1).

## Discussion

The use of accurate and high-performance bioinformatics tools to predict secreted proteins could significantly favor the search for new antituberculous vaccine candidates and drug targets, since it is well known that envelope components and surface proteins are involved in mycobacterial invasion to host cells and that secreted proteins participate in different cellular processes such as enzymatic, receptor and signal transduction mechanisms [21-23].

For any type of predictive tool, the prime interest is for it to perform well on novel data that have not been used in the process of constructing it. On the other hand, the actual predictive accuracy is intimately related to the degree of similarity existing between the training and test sets, being often relevant to measure the prediction accuracy at different levels [24]. The aim of the present study was to compare the ability of general localization and feature-based tools, to predict secreted proteins belonging to a specific biological group.

It should be highlighted that validating generic tools through the use of specific data sets allows bioinformatics users to establish the actual predictive value of the available tools. In this regard, Klee [2] and Gardy [1] reported that validation in Gram-positive proteins is a complex exercise due to the scarcity of available data to perform such type of approaches.

Validations are generally performed on generic protein sets comprising a wide range of biological species; here, we focused our validation on mycobacterial proteins. Even though we are aware that reports known to date establish a 25% identity and, particularly, that the method or methods used to obtain such estimation are not shown by the authors, we consider that the approach followed in this study is conservative, similar to approaches followed in other studies [25].

The tools included in this benchmarking analysis shared the common methodological feature that all were trained on Gram-positive protein sequences, which is highly convenient for our purposes. Though there are a large number of high-performance prediction tools, a good starting point for analyzing a biological problem is validating these tools with a novel set of previously tagged proteins.

There are also other web-available tools for predicting subcellular localization such as CELLO [26], P-CLASSIFIER [27], and BaCelLo [28], but these tools were not included in this study given that they were only trained on Gram-negative bacterial proteins (even though the latter tool also allows predicting Gram-positive bacterial proteins). Moreover, only tools based on probability theory, machine-learning skills and whose training sets are available on the web were included in this benchmarking analysis (e.g. SignalP version 3.0 was not included in this study because its training set is not yet publicly available).

On the other hand, understanding general localization tools can be of great value if their predictive capacity is known *a priori*. Likewise, users can refine their predictions by using feature-based tools, whose results provide more information regarding the tool's specificity. The ideal predictive system would be the one allowing users to combine different predictors adjustable along the decision-making process, being decision trees an example of such type of systems [13].

The tools included in this study use different prediction strategies. Particularly, PA-SUB 2.5 predictions are based on homology comparison through BLASTP against the SWISS-PROT database, while PSORTb v.2.0.4 prediction strategy combines six different modules: 1) SCL-BLAST, which is a manually curated data base , 2) Prosite motif-based analysis, 3) HMMTOP, 4) a novel outer membrane protein motif analysis, 5) SubLoc, which is a type II secretion signal peptide predictor and 6) signal peptides [29]. Accordingly, we consider that even though the tools use different methodological approaches, we believe that an appropriate way of evidencing their predictive capacities is by performing validations such as the one done in this study. We chose not to validate the predictive ability of TBpred in order to eliminate statistical bias, since the benchmarking set used in this study was derived from this tool.

In general, all tools validated in this study displayed a good performance for detecting TNs as well as FPs. The validation metrics obtained for the SignalP 2.0, TatP 1.0 and LipoP 1.0 combination presented no greater variation compared the ones obtained for SignalP 2.0 alone. This suggests that the first result depended largely upon SignalP 2.0 prediction, thereby leading us to conclude that although SignalP 2.0 had lower predictive precision than general localization tools, its large number of FNs can be explained by the fact that proteins are secreted through multiple secretory mechanisms, not all recognized by this tool, but that are possibly included within non-classical secretory pathways.

Between SignalP 2.0 and Phobius, the one showing the highest predictive capacity (in terms of validation metrics) was SignalP 2.0, which differs from the results obtained by comparing it against all the remaining feature-based tools. Regarding Signal 2.0 predictive precision, it should be noted that the results might be biased by this tool's inability to discern between a signal peptide and a transmembrane helix located at the protein's N-terminus, which suggests that Phobius might have even larger specificity and sensitivity values that would increase its MCC. Furthermore, these tools have been outlined due to their confidence and precision in most validations carried out by other authors [1,4,30], but in our analysis such metrics were below expected values.

General subcellular localization algorithms are not meant to determine by which pathway a protein is secreted, but instead to establish their specific localization, i.e., these algorithms do not yield a signal peptide presence or absence probability, but instead locate the protein in a specific subcellular compartment.

Interestingly, PA-SUB 2.5 was the best-performing prediction tool among all tools evaluated in this study, as shown by its specificity, sensitivity and MCC equal to 1. This tool had the largest training data set (10,029 sequences), which contained 43 of the 68 proteins that were removed from the analysis because they were also present within the test set (see additional file 2).

However, the outstanding performance shown by PA-SUB 2.5 can be attributed to the fact that the 272-validation protein set is included in the SWISS-PROT database, against which PA-SUB 2.5 carries out a BLAST search and builds homology profiles for each query protein. Hence, this strategy explains why all proteins were accurately recognized and classified by PA-SUB 2.5.

The expected performance of PA-SUB 2.5 with proteins displaying low similarity values than those available in SWISS-PROT is uncertain; additional studies validating prediction tools with independent data sets not included in this database and using the same approach followed in this study would provide evidence to further elucidate this issue.

According to the results, GposPLoc was the best-performing tool among those not using similarity searches against SWISS-PROT, given its high specificity and sensitivity values and its correct classification of almost all proteins in the validation set.

In general, a positive relationship was evidenced between the behavior shown by the feature-based and general localization tools in terms of specificity, while the same relationship was not observed in terms of sensitivity. Furthermore, feature-based tools presented a moderate predictive performance compared to general localization tools, given that the three tools yielded sensitivity values close to 1 (maximum value). On the basis of such evidence, it can be concluded that the feature-based tools' prediction failure is basically due to the recognition of FNs.

Despite the large number of different machine-learning tools available online, it is important for users to choose tools whose protein training sets are known as well as the mathematical and statistical methodology followed in their training, so as to recognize their strengths and weaknesses.

The protein data set used in the training of SignalP 2.0 and TatP 1.0 included both Gram-positive and Gram-negative bacterial proteins, while the training set of Lipo 1.0 included only Gram-negative bacterial proteins. However, Juncker *et al*. [11] affirmed having included a set of Gram-positive bacterial proteins in the validation of this tool, which allows it to recognize a variety of Gram-positive bacterial proteins and hence mycobacterial proteins with a 92.9% sensitivity.

According to the analysis performed in this study, none of the proteins included within the test set was detected as being a lipoprotein even though 22 proteins are annotated as being "putative lipoprotein", "lipoprotein", "pro-lipoprotein" or "uncharacterized lipoprotein" (none of them being detected as non-secreted). This result can be directly affected by the inherent complexity of training a model with sequences belonging to specific biological groups, which would hence indicate that the validation performed by Bendtsen *et al*. [10] is indeed an isolated case and that caution should be exercised when using this type of tools with biological groups different to the ones included in the training process.

Only five of the 9 proteins identified as being secreted by TatP 1.0 were also detected by SignalP 2.0. However, this result could be biased given that proteins recognized by these two prediction tools are cleaved by an SPI despite being secreted through different pathways. Interestingly, the remaining four proteins not being identified by SignalP 2.0 as secreted might correspond to proteins carrying the Tat motif that are exported to either the periplasmatic space or the extracellular milieu, irrespectively of whether there is a signal peptide or not.

In several occasions, general localization tools can place proteins in more than one subcellular localization or label them as unknown according to the prediction being applied. In the particular case of PSORTb v.2.0.4 prediction for the 272 proteins, three proteins were predicted as having multiple localization sites and 55 as "unknown". Among these proteins, 44 belonged to the set of secreted proteins while the remaining proteins were classified as being non-secreted proteins (see additional file 1). Due to the "unknown" category, the statistical result of PSORTb v.2.0.4 cannot be contrasted against the results yielded by the other tools.

## Conclusion

Altogether the results indicate that both general localization and feature-based tools had high predictive specificity and high recognition of TNs for the set of tested mycobacterial proteins. According to the results of the validation analysis, PSORTb v.2.0.4 showed higher specificity, sensitivity and MCC values than Gpos-PLoc, but failed to classify 56 proteins. Gpos-PLoc had the best predictive performance within the first approach given that it only left one protein unclassified, while SignalP 2.0 was the best one in the second approach. Even though PA-SUB 2.5 yielded the highest metrics (specificity = 1.0, sensitivity = 1.0 and MCC = 1.0), it should be taken into account that all proteins included in SWISS-PROT are used in the training process of this tool, as is the case of the protein set used in this study, either as a BLAST search or as training model. In consequence, PA-SUB 2.5 might not classify with the same accuracy a protein that is not included in SWISS-PROT.

On the other hand, the SignalP 2.0, TatP 1.0 and LipoP 1.0 combination had a similar performance to the one obtained by SignalP 2.0 alone. Therefore, it is likely that this result depended largely on SignalP 2.0.

It is important to understand which methodological strategies are used by subcellular localization prediction tools as well as to know on which protein sets they were trained, given that this allows identifying their limitations and facilitates the correct interpretation of the results. Even though new and specific prediction tools are continuously being trained, their ability to determine protein localization does not necessarily exceed that of existing general predictors. We consider that validating general predictors with specific data sets is as important as developing new specific tools.

Finally, the validation of heterogeneous tools allows users to contrast different bioinformatics methodologies under a single model. This work constitutes an important contribution to the pre-selection of target antigens for the development of new drugs and more efficient vaccines against tuberculosis.

## Methods

### Data set

The mycobacterial protein sequence data set used for validating the different prediction tools was obtained from the training set of the TBpred tool [17], which is an algorithm specifically designed for predicting subcellular localization of mycobacterial proteins. This tool's data set contains 852 proteins (extracted from SWISS-PROT), which were classified by Mamoon *et al*. into four major localization classes, each containing a reasonable number of proteins, as follows: 340 cyt, 402 imp, 50 sec and 60 amla proteins [17]. In this work, the entire sequence set was filtered by using the Cd-hit algorithm [16] in order to obtain a final test set containing only proteins having less than 40% identity and hence discard redundant sequences.

As a result of this preliminary analysis, a total of 340 proteins was obtained. However, when these proteins were compared to the sequences of each tool's training data set, 68 proteins were shared between both data sets (see additional file 2). Therefore, in order to eliminate protein redundancy in the validation data set, these shared proteins were not considered within the final test set.

Each protein's annotation was retrieved from the NCBI database by using the blastp tool (available at ). In addition, the results of the subcellular localization tools and the sequences of the 204 secreted and 68 non-secreted proteins are provided together with this article in additional files 1 and 3, respectively.

### Categorizing the validation set

The validation set was divided into two groups: 1) Secreted proteins (n = 204), corresponding to imp, amla and sec; and 2) non-secreted proteins (n = 68), including all cyto proteins.

### Criteria for selecting bioinformatics tools

The following criteria were chosen for selecting the bioinformatics tools considered in this study: 1) having training data sets available to the general public, 2) being trained with Gram-positive bacterial protein sequences, except for TatP 1.0 (even though Bendtsen *et al*. proved its predictive efficacy on an artificial set of Gram-positive bacterial proteins [10]), and 3) being based on probability theory and built as machine-learning methods.

The predictive tools used two methods, general localization and the feature-based approach [1]. The first one was oriented towards defining the proteins' subcellular localization by using the Protein Analyst-homology-based subcellular localization predictor (PA-SUB server v2.5) [4,31] hosted at , Gpos-PLoc [13] at  and PSORTb v.2.0.4 [29] at .

The second approach used SignalP 2.0 [9] which recognized type I signal peptides , TatP 1.0 [10] which detects Tat-transporter signal peptides , LipoP 1.0 [11] that identifies type II signal peptides (lipoproteins)  and Phobius [12] that allows identifying type I and II signal peptides. Table 2 shows the features related to subcellular localization prediction recognized by each of the different tools.

**Table 2:** Protein sequence features related to subcellular localization prediction.

	**Secretion System and/or SP**									
**TOOLS**	**SPI**	**SPII**	**Cyt**	**TM**	**Type I**	**Type II**	**Cell Wall**	**Extracell**	**Plasma membrane**	**Periplasm**
SignalP 2.0 (Sec-dependent)	X							X	X	
TatP 1.0 (altern system)	X						X	X	X	
LipoP 1.0 (Sec-dependent)	X	X	X	X				X	X	
Phobius				X	X	X				
PA-SUB 2.5	X	X	X							
Gpos-Ploc			X				X	X	X	X
PSORTb v.2.0.4			X					X	X	X

**SP**, Signal Peptidase. **SPI**, Signal Peptidase I. **SPII**, Signal Peptidase II. **Cyt**, Cytoplasmatic. **TM**, Transmembranal protein.

### Threshold values for feature-based and general localization tools

The threshold for the feature-based tools SignalP 2.0 (HMM-calculated probability of having a signal peptide) and TatP 1.0 (max. S value) was set to ≥ 0.5 score. As it can be observed in the additional file 1, only those proteins showing a characteristic Tat motif within their signal peptides were further used for the detailed metrics evaluation. The best observed score for the probability of having a SPase II cleavage site was used as the threshold for LipoP 1.0. In the case of Phobius, only proteins predicted as having a signal peptide (denoted by the letter Y) were included in the analysis.

Regarding general localization tools, all those proteins predicted by PA-SUB 2.5 as being extracellular or plasma membrane proteins, as well as those predicted by PSORTb v.2.0.4 as being extracellularly located or on the cytoplasmatic membrane, and those classified by Gpos-Ploc as extracellular or plasma membrane proteins were included in the analysis.

### Metric evaluation

The application of consistent metrics for validating the different tools involves some difficulties such as analyzing data under the same standardized criteria and the bias resulting from the use of ideal data sets that lead to overrating the tools predictive value [24,30].

The tools were validated based on current statistical techniques applying the following parameters: i) a confusion matrix which involved classifying tool's predictions within the following categories: 1) TPs, 2) TNs, 3) FPs and 4) FNs, and whose criteria were set *a priori *based on the categorization of the validation set (see Table 3). ii) Quality considered as the accuracy of the prediction beyond quantity (number of predicted proteins), which was established by using a measure known as precision or specificity that was calculated as TPs/(TPs+FPs). iii) The prediction estimate was coupled with a sensitivity measure reflecting each tool's capacity to identify TPs, computed as TPs/(TPs+FNs) [1,2,32].

**Table 3:** Criteria used for constructing the confusion matrix.

**Parameters**	**Secreted Category**
**TPs**	Protein predicted as secreted being secreted.
**TNs**	Protein predicted as non-secreted being non-secreted.
**FPs**	Protein predicted as secreted being non-secreted.
**FNs**	Protein predicted as non-secreted being secreted.

Additionally, the precision of each method was established based on the MCC [33], which ranges between -1 and +1 and can be used on non-binary variables. According to MCC values, a -1 value indicates an inaccurate prediction while a +1 value denotes an accurate prediction and 0 denotes a random prediction [24]. MCC values were computed using the following formula [1]:



## Authors' contributions

DR-M and CV wrote the manuscript, validated the tools and carried out the data analysis and interpretation. LFN, MO, MEP and MAP contributed to the methodological design, supervised its development and critically revised the manuscript's content. MAP supervised the research group. All authors read and approved the final version of the manuscript.

## Supplementary Material

Additional file 1 **Results of subcellular localization prediction tools**. Subcellular localization prediction obtained with each tool and construction of the confusion matrix. Click here for file

Additional file 2 **Proteins shared between prediction tools**. Common proteins identified within the training databases of each validated prediction tool. Click here for file

Additional file 3 **Protein validation data set**. Fasta sequences of the proteins used validating subcellular localization prediction tool. Click here for file
